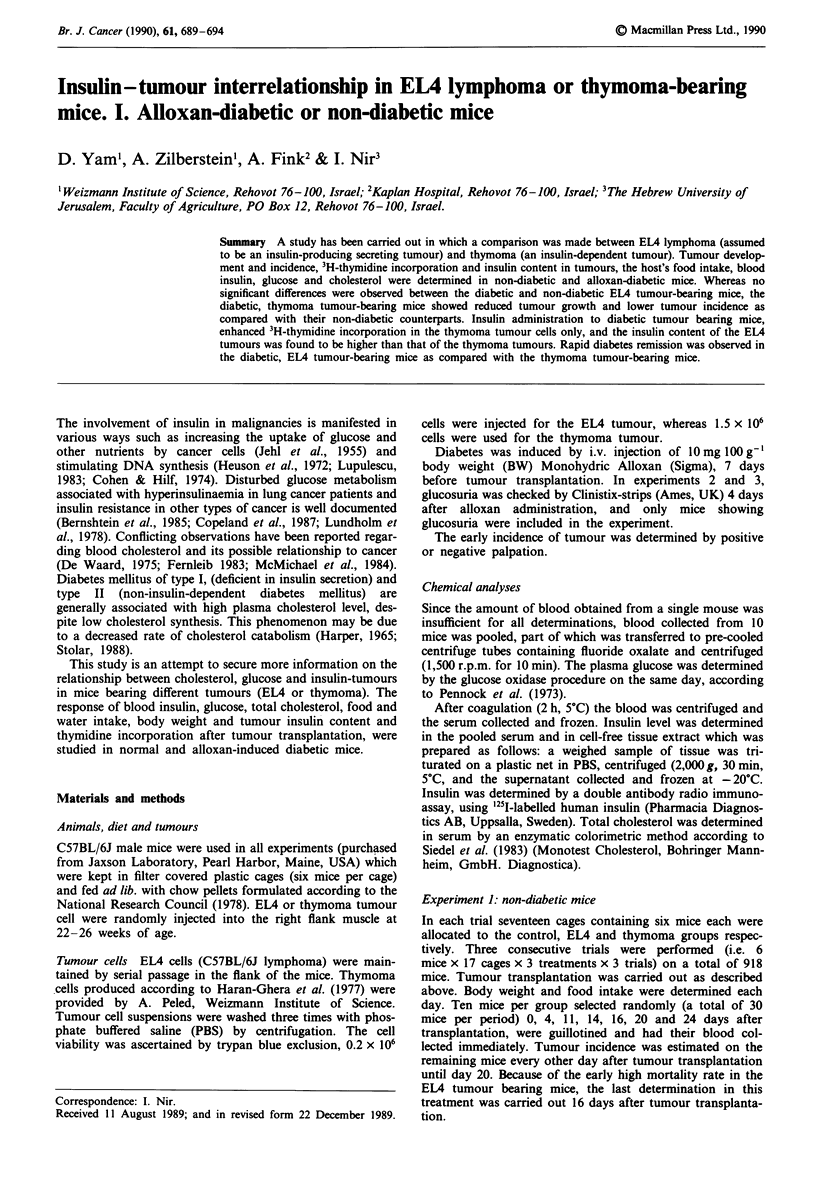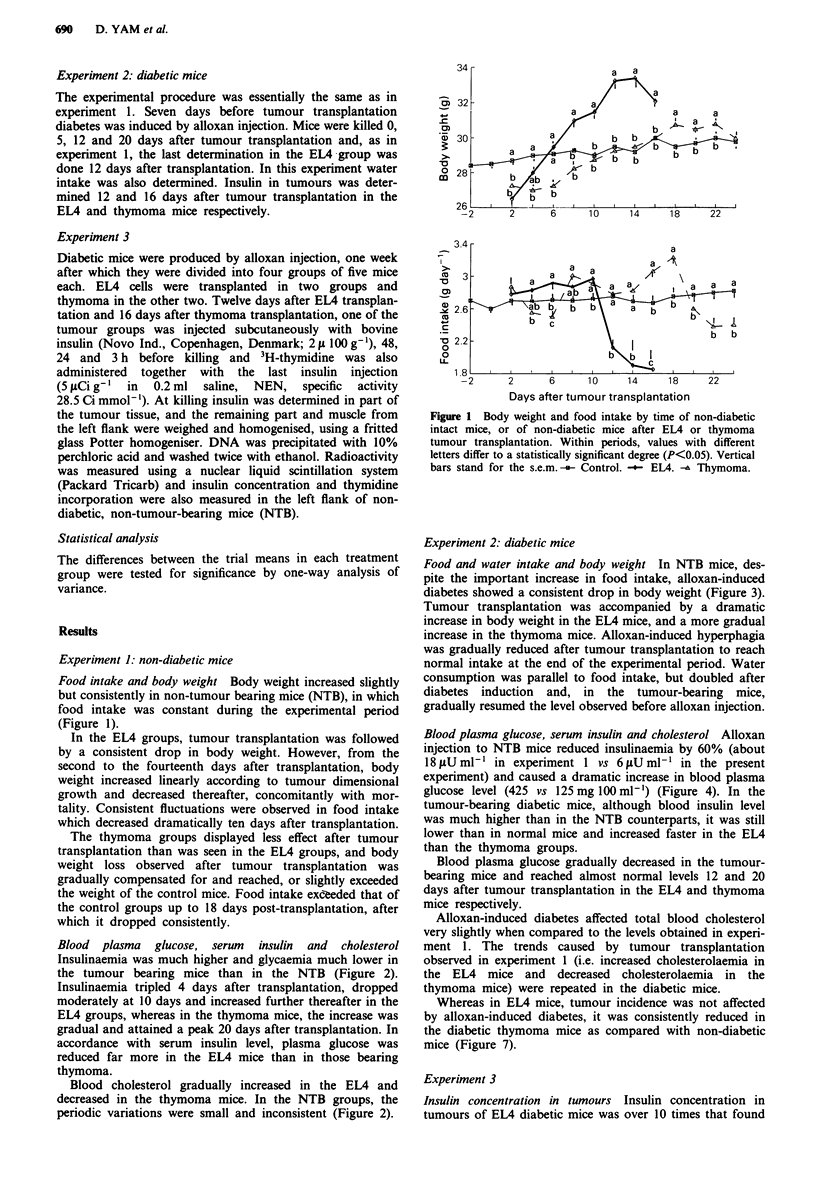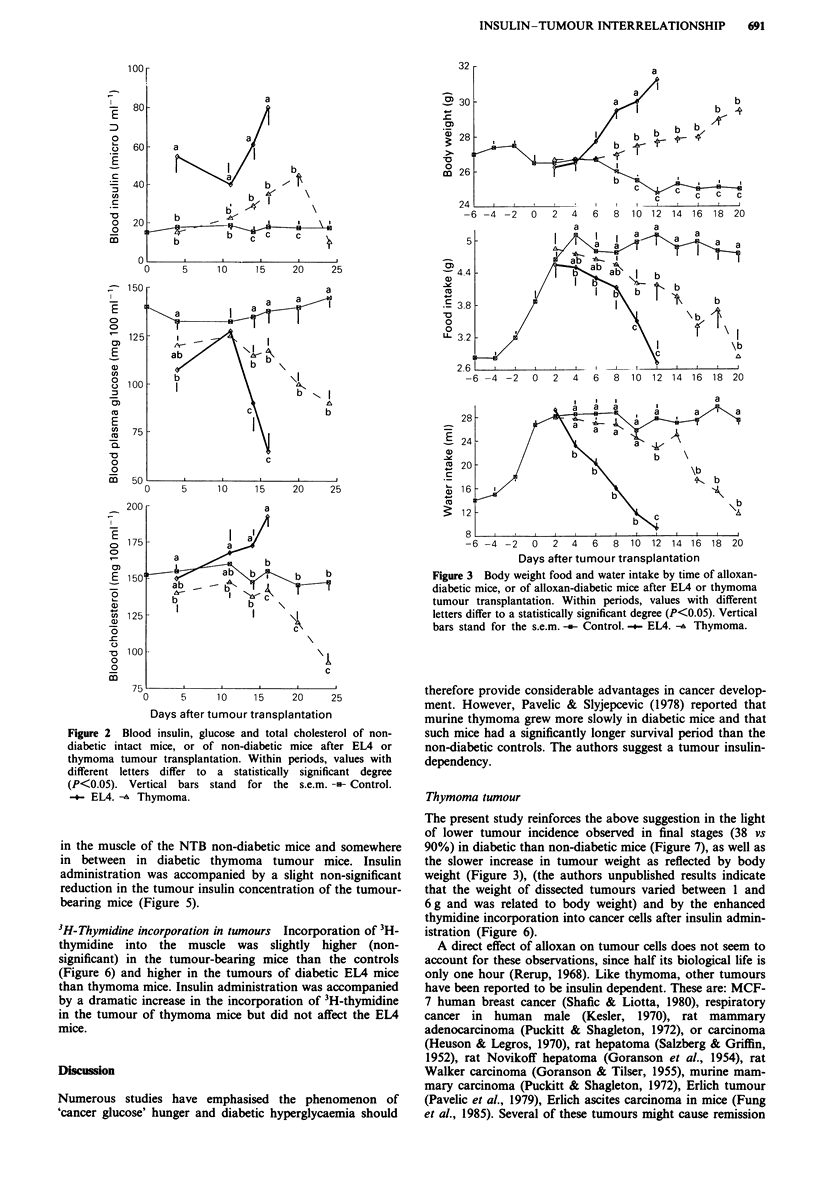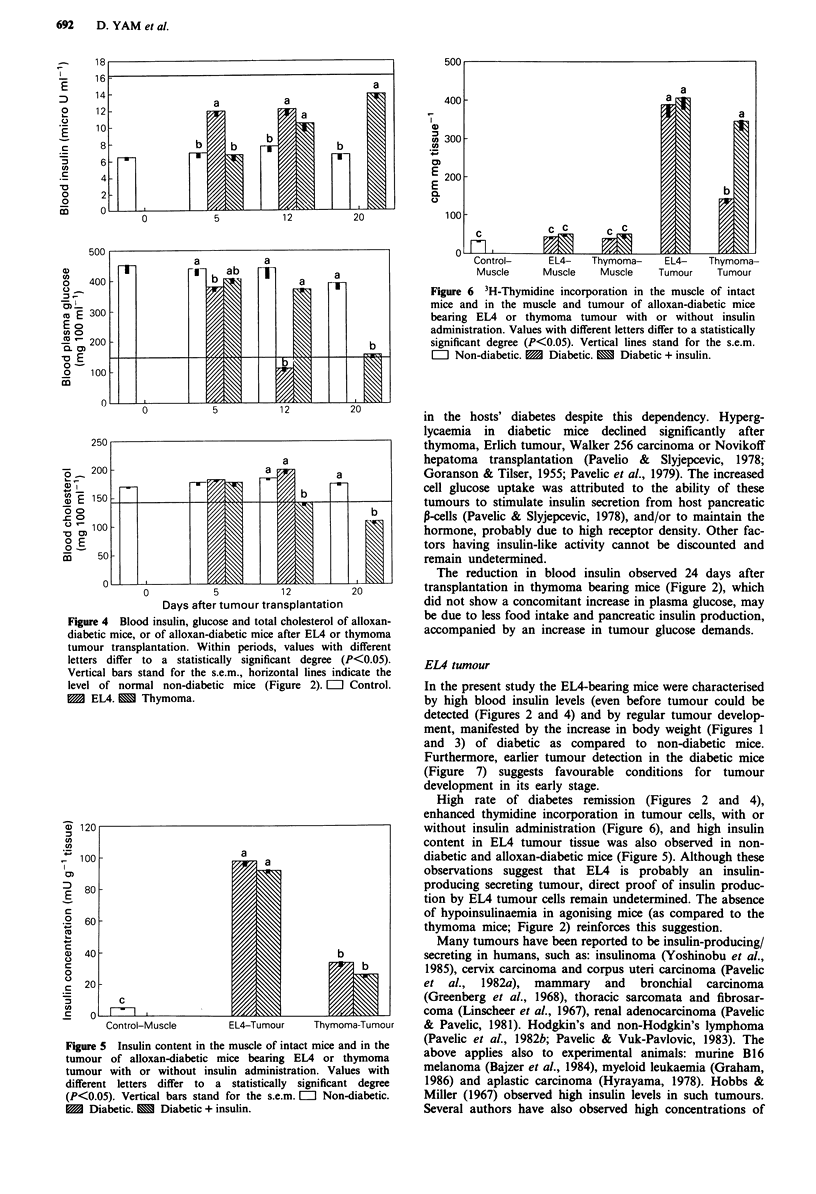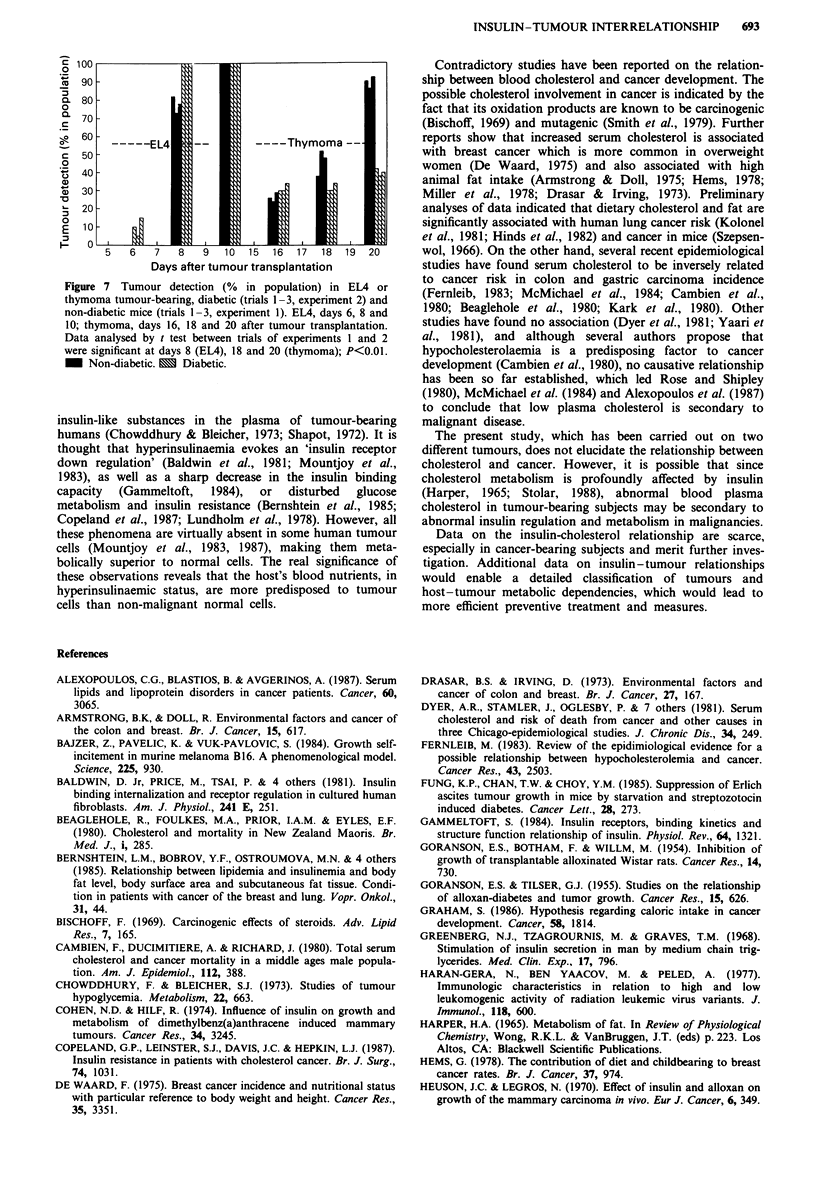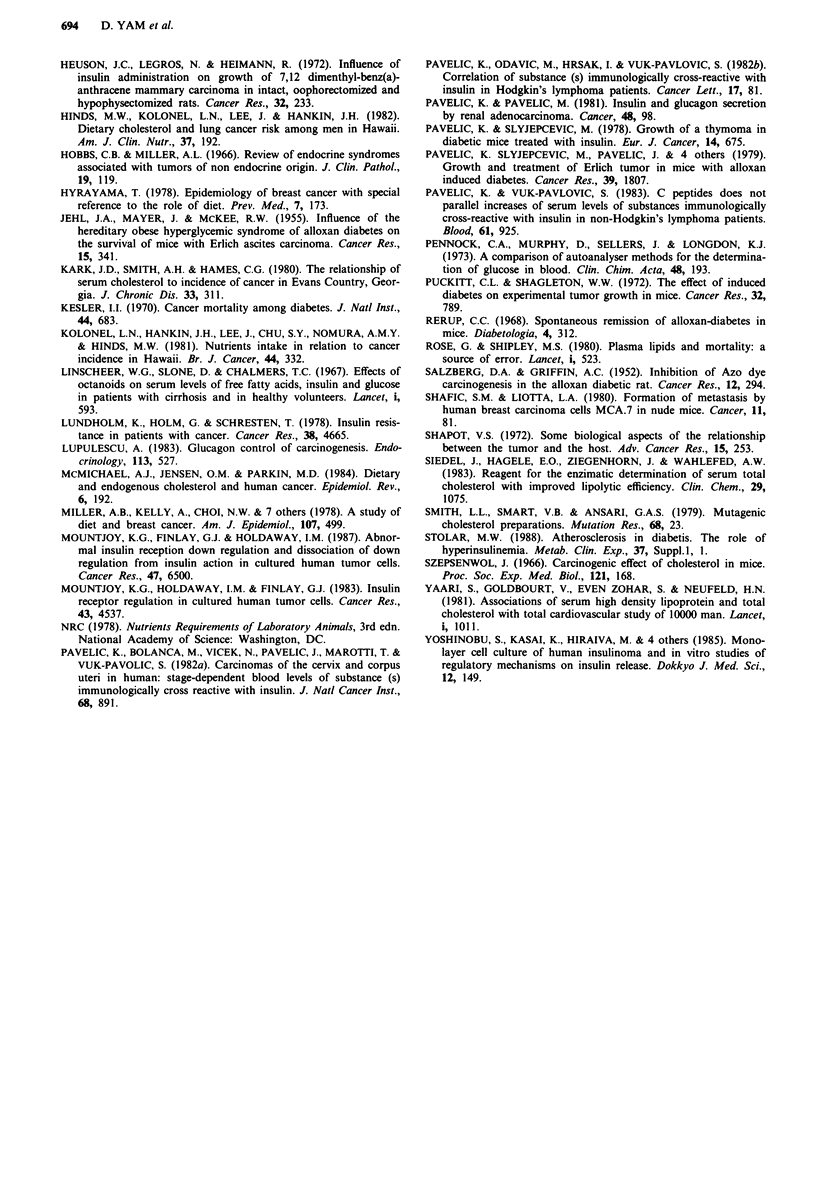# Insulin-tumour interrelationship in EL4 lymphoma or thymoma-bearing mice. I. Alloxan-diabetic or non-diabetic mice.

**DOI:** 10.1038/bjc.1990.156

**Published:** 1990-05

**Authors:** D. Yam, A. Zilberstein, A. Fink, I. Nir

**Affiliations:** Weizmann Institute of Science, Rehovot, Israel.

## Abstract

A study has been carried out in which a comparison was made between EL4 lymphoma (assumed to be an insulin-producing secreting tumour) and thymoma (an insulin-dependent tumour). Tumour development and incidence, 3H-thymidine incorporation and insulin content in tumours, the host's food intake, blood insulin, glucose and cholesterol were determined in non-diabetic and alloxan-diabetic mice. Whereas no significant differences were observed between the diabetic and non-diabetic EL4 tumour-bearing mice, the diabetic, thymoma tumour-bearing mice showed reduced tumour growth and lower tumour incidence as compared with their non-diabetic counterparts. Insulin administration to diabetic tumour bearing mice, enhanced 3H-thymidine incorporation in the thymoma tumour cells only, and the insulin content of the EL4 tumours was found to be higher than that of the thymoma tumours. Rapid diabetes remission was observed in the diabetic, EL4 tumour-bearing mice as compared with the thymoma tumour-bearing mice.


Br. J. Cancer (1990), 61, 689-694 © Macmillan Press Ltd., 1990

### Insulin-tumour interrelationship in EL4 lymphoma or thymoma-bearing mice. I. Alloxan-diabetic or non-diabetic mice

D. Yam[1], A. Zilberstein[1], A. Fink[2] & I. Nir[3]

[1]Weizmann Institute of Science, Rehovot 76-100, Israel; [2]Kaplan Hospital, Rehovot 76-100, Israel; [3]The Hebrew University of Jerusalem, Faculty of Agriculture, PO Box 12, Rehovot 76-100, Israel.

**Summary** A study has been carried out in which a comparison was made between EL4 lymphoma (assumed to be an insulin-producing secreting tumour) and thymoma (an insulin-dependent tumour). Tumour development and incidence, $^3$H-thymidine incorporation and insulin content in tumours, the host's food intake, blood insulin, glucose and cholesterol were determined in non-diabetic and alloxan-diabetic mice. Whereas no significant differences were observed between the diabetic and non-diabetic EL4 tumour-bearing mice, the diabetic, thymoma tumour-bearing mice showed reduced tumour growth and lower tumour incidence as compared with their non-diabetic counterparts. Insulin administration to diabetic tumour bearing mice, enhanced $^3$H-thymidine incorporation in the thymoma tumour cells only, and the insulin content of the EL4 tumours was found to be higher than that of the thymoma tumours. Rapid diabetes remission was observed in the diabetic, EL4 tumour-bearing mice as compared with the thymoma tumour-bearing mice.

The involvement of insulin in malignancies is manifested in various ways such as increasing the uptake of glucose and other nutrients by cancer cells (Jehl *et al.*, 1955) and stimulating DNA synthesis (Heuson *et al.*, 1972; Lupulescu, 1983; Cohen & Hilf, 1974). Disturbed glucose metabolism associated with hyperinsulinaemia in lung cancer patients and insulin resistance in other types of cancer is well documented (Bernshtein *et al.*, 1985; Copeland *et al.*, 1987; Lundholm *et al.*, 1978). Conflicting observations have been reported regarding blood cholesterol and its possible relationship to cancer (De Waard, 1975; Fernleib 1983; McMichael *et al.*, 1984). Diabetes mellitus of type I, (deficient in insulin secretion) and type II (non-insulin-dependent diabetes mellitus) are generally associated with high plasma cholesterol level, despite low cholesterol synthesis. This phenomenon may be due to a decreased rate of cholesterol catabolism (Harper, 1965; Stolar, 1988).

This study is an attempt to secure more information on the relationship between cholesterol, glucose and insulin-tumours in mice bearing different tumours (EL4 or thymoma). The response of blood insulin, glucose, total cholesterol, food and water intake, body weight and tumour insulin content and thymidine incorporation after tumour transplantation, were studied in normal and alloxan-induced diabetic mice.

### Materials and methods

### *Animals, diet and tumours*

C57BL/6J male mice were used in all experiments (purchased from Jaxson Laboratory, Pearl Harbor, Maine, USA) which were kept in filter covered plastic cages (six mice per cage) and fed *ad lib.* with chow pellets formulated according to the National Research Council (1978). EL4 or thymoma tumour cell were randomly injected into the right flank muscle at 22-26 weeks of age.

*Tumour cells* EL4 cells (C57BL/6J lymphoma) were maintained by serial passage in the flank of the mice. Thymoma cells produced according to Haran-Ghera *et al.* (1977) were provided by A. Peled, Weizmann Institute of Science. Tumour cell suspensions were washed three times with phosphate buffered saline (PBS) by centrifugation. The cell viability was ascertained by trypan blue exclusion, $0.2 \times 10^6$ cells were injected for the EL4 tumour, whereas $1.5 \times 10^6$ cells were used for the thymoma tumour.

Diabetes was induced by i.v. injection of 10 mg 100 g$^{-1}$ body weight (BW) Monohydric Alloxan (Sigma), 7 days before tumour transplantation. In experiments 2 and 3, glucosuria was checked by Clinistix-strips (Ames, UK) 4 days after alloxan administration, and only mice showing glucosuria were included in the experiment.

The early incidence of tumour was determined by positive or negative palpation.

### *Chemical analyses*

Since the amount of blood obtained from a single mouse was insufficient for all determinations, blood collected from 10 mice was pooled, part of which was transferred to pre-cooled centrifuge tubes containing fluoride oxalate and centrifuged (1,500 r.p.m. for 10 min). The plasma glucose was determined by the glucose oxidase procedure on the same day, according to Pennock *et al.* (1973).

After coagulation (2 h, 5°C) the blood was centrifuged and the serum collected and frozen. Insulin level was determined in the pooled serum and in cell-free tissue extract which was prepared as follows: a weighed sample of tissue was triturated on a plastic net in PBS, centrifuged (2,000 ***g***, 30 min, 5°C, and the supernatant collected and frozen at $-20$°C. Insulin was determined by a double antibody radio immunoassay, using $^{125}$I-labelled human insulin (Pharmacia Diagnostics AB, Uppsalla, Sweden). Total cholesterol was determined in serum by an enzymatic colorimetric method according to Siedel *et al.* (1983) (Monotest Cholesterol, Bohringer Mannheim, GmbH. Diagnostica).

### *Experiment 1: non-diabetic mice*

In each trial seventeen cages containing six mice each were allocated to the control, EL4 and thymoma groups respectively. Three consecutive trials were performed (i.e. 6 mice × 17 cages × 3 treatments × 3 trials) on a total of 918 mice. Tumour transplantation was carried out as described above. Body weight and food intake were determined each day. Ten mice per group selected randomly (a total of 30 mice per period) 0, 4, 11, 14, 16, 20 and 24 days after transplantation, were guillotined and had their blood collected immediately. Tumour incidence was estimated on the remaining mice every other day after tumour transplantation until day 20. Because of the early high mortality rate in the EL4 tumour bearing mice, the last determination in this treatment was carried out 16 days after tumour transplantation.

Correspondence: I. Nir.
Received 11 August 1989; and in revised form 22 December 1989.

*Experiment 2: diabetic mice*

The experimental procedure was essentially the same as in experiment 1. Seven days before tumour transplantation diabetes was induced by alloxan injection. Mice were killed 0, 5, 12 and 20 days after tumour transplantation and, as in experiment 1, the last determination in the EL4 group was done 12 days after transplantation. In this experiment water intake was also determined. Insulin in tumours was determined 12 and 16 days after tumour transplantation in the EL4 and thymoma mice respectively.

*Experiment 3*

Diabetic mice were produced by alloxan injection, one week after which they were divided into four groups of five mice each. EL4 cells were transplanted in two groups and thymoma in the other two. Twelve days after EL4 transplantation and 16 days after thymoma transplantation, one of the tumour groups was injected subcutaneously with bovine insulin (Novo Ind., Copenhagen, Denmark; 2 $\mu$ 100 $g^{-1}$), 48, 24 and 3 h before killing and $^3$H-thymidine was also administered together with the last insulin injection (5 $\mu Ci\ g^{-1}$ in 0.2 ml saline, NEN, specific activity 28.5 Ci $mmol^{-1}$). At killing insulin was determined in part of the tumour tissue, and the remaining part and muscle from the left flank were weighed and homogenised, using a fritted glass Potter homogeniser. DNA was precipitated with 10% perchloric acid and washed twice with ethanol. Radioactivity was measured using a nuclear liquid scintillation system (Packard Tricarb) and insulin concentration and thymidine incorporation were also measured in the left flank of non-diabetic, non-tumour-bearing mice (NTB).

*Statistical analysis*

The differences between the trial means in each treatment group were tested for significance by one-way analysis of variance.

### Results

*Experiment 1: non-diabetic mice*

*Food intake and body weight* Body weight increased slightly but consistently in non-tumour bearing mice (NTB), in which food intake was constant during the experimental period (Figure 1).

In the EL4 groups, tumour transplantation was followed by a consistent drop in body weight. However, from the second to the fourteenth days after transplantation, body weight increased linearly according to tumour dimensional growth and decreased thereafter, concomitantly with mortality. Consistent fluctuations were observed in food intake which decreased dramatically ten days after transplantation.

The thymoma groups displayed less effect after tumour transplantation than was seen in the EL4 groups, and body weight loss observed after tumour transplantation was gradually compensated for and reached, or slightly exceeded the weight of the control mice. Food intake exceeded that of the control groups up to 18 days post-transplantation, after which it dropped consistently.

*Blood plasma glucose, serum insulin and cholesterol* Insulinaemia was much higher and glycaemia much lower in the tumour bearing mice than in the NTB (Figure 2). Insulinaemia tripled 4 days after transplantation, dropped moderately at 10 days and increased further thereafter in the EL4 groups, whereas in the thymoma mice, the increase was gradual and attained a peak 20 days after transplantation. In accordance with serum insulin level, plasma glucose was reduced far more in the EL4 mice than in those bearing thymoma.

Blood cholesterol gradually increased in the EL4 and decreased in the thymoma mice. In the NTB groups, the periodic variations were small and inconsistent (Figure 2).

**Figure 1** Body weight and food intake by time of non-diabetic intact mice, or of non-diabetic mice after EL4 or thymoma tumour transplantation. Within periods, values with different letters differ to a statistically significant degree ($P<0.05$). Vertical bars stand for the s.e.m. –■– Control. –◆– EL4. –▲ Thymoma.

*Experiment 2: diabetic mice*

*Food and water intake and body weight* In NTB mice, despite the important increase in food intake, alloxan-induced diabetes showed a consistent drop in body weight (Figure 3). Tumour transplantation was accompanied by a dramatic increase in body weight in the EL4 mice, and a more gradual increase in the thymoma mice. Alloxan-induced hyperphagia was gradually reduced after tumour transplantation to reach normal intake at the end of the experimental period. Water consumption was parallel to food intake, but doubled after diabetes induction and, in the tumour-bearing mice, gradually resumed the level observed before alloxan injection.

*Blood plasma glucose, serum insulin and cholesterol* Alloxan injection to NTB mice reduced insulinaemia by 60% (about 18 $\mu U\ ml^{-1}$ in experiment 1 *vs* 6 $\mu U\ ml^{-1}$ in the present experiment) and caused a dramatic increase in blood plasma glucose level (425 *vs* 125 mg 100 $ml^{-1}$) (Figure 4). In the tumour-bearing diabetic mice, although blood insulin level was much higher than in the NTB counterparts, it was still lower than in normal mice and increased faster in the EL4 than the thymoma groups.

Blood plasma glucose gradually decreased in the tumour-bearing mice and reached almost normal levels 12 and 20 days after tumour transplantation in the EL4 and thymoma mice respectively.

Alloxan-induced diabetes affected total blood cholesterol very slightly when compared to the levels obtained in experiment 1. The trends caused by tumour transplantation observed in experiment 1 (i.e. increased cholesterolaemia in the EL4 mice and decreased cholesterolaemia in the thymoma mice) were repeated in the diabetic mice.

Whereas in EL4 mice, tumour incidence was not affected by alloxan-induced diabetes, it was consistently reduced in the diabetic thymoma mice as compared with non-diabetic mice (Figure 7).

*Experiment 3*

*Insulin concentration in tumours* Insulin concentration in tumours of EL4 diabetic mice was over 10 times that found

**Figure 2** Blood insulin, glucose and total cholesterol of non-diabetic intact mice, or of non-diabetic mice after EL4 or thymoma tumour transplantation. Within periods, values with different letters differ to a statistically significant degree ($P<0.05$). Vertical bars stand for the s.e.m. -■- Control. -◆- EL4. -▲ Thymoma.

in the muscle of the NTB non-diabetic mice and somewhere in between in diabetic thymoma tumour mice. Insulin administration was accompanied by a slight non-significant reduction in the tumour insulin concentration of the tumour-bearing mice (Figure 5).

*$^3$H-Thymidine incorporation in tumours* Incorporation of $^3$H-thymidine into the muscle was slightly higher (non-significant) in the tumour-bearing mice than the controls (Figure 6) and higher in the tumours of diabetic EL4 mice than thymoma mice. Insulin administration was accompanied by a dramatic increase in the incorporation of $^3$H-thymidine in the tumour of thymoma mice but did not affect the EL4 mice.

### Discussion

Numerous studies have emphasised the phenomenon of 'cancer glucose' hunger and diabetic hyperglycaemia should

**Figure 3** Body weight food and water intake by time of alloxan-diabetic mice, or of alloxan-diabetic mice after EL4 or thymoma tumour transplantation. Within periods, values with different letters differ to a statistically significant degree ($P<0.05$). Vertical bars stand for the s.e.m. -■- Control. -◆- EL4. -▲ Thymoma.

therefore provide considerable advantages in cancer development. However, Pavelic & Slyjepcevic (1978) reported that murine thymoma grew more slowly in diabetic mice and that such mice had a significantly longer survival period than the non-diabetic controls. The authors suggest a tumour insulin-dependency.

### *Thymoma tumour*

The present study reinforces the above suggestion in the light of lower tumour incidence observed in final stages (38 *vs* 90%) in diabetic than non-diabetic mice (Figure 7), as well as the slower increase in tumour weight as reflected by body weight (Figure 3), (the authors unpublished results indicate that the weight of dissected tumours varied between 1 and 6 g and was related to body weight) and by the enhanced thymidine incorporation into cancer cells after insulin administration (Figure 6).

A direct effect of alloxan on tumour cells does not seem to account for these observations, since half its biological life is only one hour (Rerup, 1968). Like thymoma, other tumours have been reported to be insulin dependent. These are: MCF-7 human breast cancer (Shafic & Liotta, 1980), respiratory cancer in human male (Kesler, 1970), rat mammary adenocarcinoma (Puckitt & Shagleton, 1972), or carcinoma (Heuson & Legros, 1970), rat hepatoma (Salzberg & Griffin, 1952), rat Novikoff hepatoma (Goranson *et al.*, 1954), rat Walker carcinoma (Goranson & Tilser, 1955), murine mammary carcinoma (Puckitt & Shagleton, 1972), Erlich tumour (Pavelic *et al.*, 1979), Erlich ascites carcinoma in mice (Fung *et al.*, 1985). Several of these tumours might cause remission

**Figure 4** Blood insulin, glucose and total cholesterol of alloxan-diabetic mice, or of alloxan-diabetic mice after EL4 or thymoma tumour transplantation. Within periods, values with different letters differ to a statistically significant degree ($P<0.05$). Vertical bars stand for the s.e.m., horizontal lines indicate the level of normal non-diabetic mice (Figure 2). ☐ Control. ▨ EL4. ▧ Thymoma.

**Figure 5** Insulin content in the muscle of intact mice and in the tumour of alloxan-diabetic mice bearing EL4 or thymoma tumour with or without insulin administration. Values with different letters differ to a statistically significant degree ($P<0.05$). Vertical bars stand for the s.e.m. ☐ Non-diabetic. ▨ Diabetic. ▧ Diabetic + insulin.

**Figure 6** $^3$H-Thymidine incorporation in the muscle of intact mice and in the muscle and tumour of alloxan-diabetic mice bearing EL4 or thymoma tumour with or without insulin administration. Values with different letters differ to a statistically significant degree ($P<0.05$). Vertical lines stand for the s.e.m. ☐ Non-diabetic. ▨ Diabetic. ▧ Diabetic + insulin.

in the hosts' diabetes despite this dependency. Hyperglycaemia in diabetic mice declined significantly after thymoma, Erlich tumour, Walker 256 carcinoma or Novikoff hepatoma transplantation (Pavelio & Slyjepcevic, 1978; Goranson & Tilser, 1955; Pavelic et al., 1979). The increased cell glucose uptake was attributed to the ability of these tumours to stimulate insulin secretion from host pancreatic β-cells (Pavelic & Slyjepcevic, 1978), and/or to maintain the hormone, probably due to high receptor density. Other factors having insulin-like activity cannot be discounted and remain undetermined.

The reduction in blood insulin observed 24 days after transplantation in thymoma bearing mice (Figure 2), which did not show a concomitant increase in plasma glucose, may be due to less food intake and pancreatic insulin production, accompanied by an increase in tumour glucose demands.

### *EL4 tumour*

In the present study the EL4-bearing mice were characterised by high blood insulin levels (even before tumour could be detected (Figures 2 and 4) and by regular tumour development, manifested by the increase in body weight (Figures 1 and 3) of diabetic as compared to non-diabetic mice. Furthermore, earlier tumour detection in the diabetic mice (Figure 7) suggests favourable conditions for tumour development in its early stage.

High rate of diabetes remission (Figures 2 and 4), enhanced thymidine incorporation in tumour cells, with or without insulin administration (Figure 6), and high insulin content in EL4 tumour tissue was also observed in non-diabetic and alloxan-diabetic mice (Figure 5). Although these observations suggest that EL4 is probably an insulin-producing secreting tumour, direct proof of insulin production by EL4 tumour cells remain undetermined. The absence of hypoinsulinaemia in agonising mice (as compared to the thymoma mice; Figure 2) reinforces this suggestion.

Many tumours have been reported to be insulin-producing/secreting in humans, such as: insulinoma (Yoshinobu et al., 1985), cervix carcinoma and corpus uteri carcinoma (Pavelic et al., 1982a), mammary and bronchial carcinoma (Greenberg et al., 1968), thoracic sarcomata and fibrosarcoma (Linscheer et al., 1967), renal adenocarcinoma (Pavelic & Pavelic, 1981). Hodgkin's and non-Hodgkin's lymphoma (Pavelic et al., 1982b; Pavelic & Vuk-Pavlovic, 1983). The above applies also to experimental animals: murine B16 melanoma (Bajzer et al., 1984), myeloid leukaemia (Graham, 1986) and aplastic carcinoma (Hyrayama, 1978). Hobbs & Miller (1967) observed high insulin levels in such tumours. Several authors have also observed high concentrations of

**Figure 7** Tumour detection (% in population) in EL4 or thymoma tumour-bearing, diabetic (trials 1–3, experiment 2) and non-diabetic mice (trials 1–3, experiment 1). EL4, days 6, 8 and 10; thymoma, days 16, 18 and 20 after tumour transplantation. Data analysed by *t* test between trials of experiments 1 and 2 were significant at days 8 (EL4), 18 and 20 (thymoma); $P<0.01$. ■ Non-diabetic. ▧ Diabetic.

insulin-like substances in the plasma of tumour-bearing humans (Chowddhury & Bleicher, 1973; Shapot, 1972). It is thought that hyperinsulinaemia evokes an 'insulin receptor down regulation' (Baldwin *et al.*, 1981; Mountjoy *et al.*, 1983), as well as a sharp decrease in the insulin binding capacity (Gammeltoft, 1984), or disturbed glucose metabolism and insulin resistance (Bernshtein *et al.*, 1985; Copeland *et al.*, 1987; Lundholm *et al.*, 1978). However, all these phenomena are virtually absent in some human tumour cells (Mountjoy *et al.*, 1983, 1987), making them metabolically superior to normal cells. The real significance of these observations reveals that the host's blood nutrients, in hyperinsulinaemic status, are more predisposed to tumour cells than non-malignant normal cells.

Contradictory studies have been reported on the relationship between blood cholesterol and cancer development. The possible cholesterol involvement in cancer is indicated by the fact that its oxidation products are known to be carcinogenic (Bischoff, 1969) and mutagenic (Smith *et al.*, 1979). Further reports show that increased serum cholesterol is associated with breast cancer which is more common in overweight women (De Waard, 1975) and also associated with high animal fat intake (Armstrong & Doll, 1975; Hems, 1978; Miller *et al.*, 1978; Drasar & Irving, 1973). Preliminary analyses of data indicated that dietary cholesterol and fat are significantly associated with human lung cancer risk (Kolonel *et al.*, 1981; Hinds *et al.*, 1982) and cancer in mice (Szepsenwol, 1966). On the other hand, several recent epidemiological studies have found serum cholesterol to be inversely related to cancer risk in colon and gastric carcinoma incidence (Fernleib, 1983; McMichael *et al.*, 1984; Cambien *et al.*, 1980; Beaglehole *et al.*, 1980; Kark *et al.*, 1980). Other studies have found no association (Dyer *et al.*, 1981; Yaari *et al.*, 1981), and although several authors propose that hypocholesterolaemia is a predisposing factor to cancer development (Cambien *et al.*, 1980), no causative relationship has been so far established, which led Rose and Shipley (1980), McMichael *et al.* (1984) and Alexopoulos *et al.* (1987) to conclude that low plasma cholesterol is secondary to malignant disease.

The present study, which has been carried out on two different tumours, does not elucidate the relationship between cholesterol and cancer. However, it is possible that since cholesterol metabolism is profoundly affected by insulin (Harper, 1965; Stolar, 1988), abnormal blood plasma cholesterol in tumour-bearing subjects may be secondary to abnormal insulin regulation and metabolism in malignancies.

Data on the insulin-cholesterol relationship are scarce, especially in cancer-bearing subjects and merit further investigation. Additional data on insulin–tumour relationships would enable a detailed classification of tumours and host–tumour metabolic dependencies, which would lead to more efficient preventive treatment and measures.

### References

ALEXOPOULOS, C.G., BLASTIOS, B. & AVGERINOS, A. (1987). Serum lipids and lipoprotein disorders in cancer patients. *Cancer*, **60**, 3065.

ARMSTRONG, B.K, & DOLL, R. Environmental factors and cancer of the colon and breast. *Br. J. Cancer*, **15**, 617.

BAJZER, Z., PAVELIC, K. & VUK-PAVLOVIC, S. (1984). Growth self-incitement in murine melanoma B16. A phenomenological model. *Science*, **225**, 930.

BALDWIN, D. Jr, PRICE, M., TSAI, P. & 4 others (1981). Insulin binding internalization and receptor regulation in cultured human fibroblasts. *Am. J. Physiol.*, **241 E**, 251.

BEAGLEHOLE, R., FOULKES, M.A., PRIOR, I.A.M. & EYLES, E.F. (1980). Cholesterol and mortality in New Zealand Maoris. *Br. Med. J.*, **i**, 285.

BERNSHTEIN, L.M., BOBROV, Y.F., OSTROUMOVA, M.N. & 4 others (1985). Relationship between lipidemia and insulinemia and body fat level, body surface area and subcutaneous fat tissue. Condition in patients with cancer of the breast and lung. *Vopr. Onkol.*, **31**, 44.

BISCHOFF, F. (1969). Carcinogenic effects of steroids. *Adv. Lipid Res.*, **7**, 165.

CAMBIEN, F., DUCIMITIERE, A. & RICHARD, J. (1980). Total serum cholesterol and cancer mortality in a middle ages male population. *Am. J. Epidemiol.*, **112**, 388.

CHOWDDHURY, F. & BLEICHER, S.J. (1973). Studies of tumour hypoglycemia. *Metabolism*, **22**, 663.

COHEN, N.D. & HILF, R. (1974). Influence of insulin on growth and metabolism of dimethylbenz(a)anthracene induced mammary tumours. *Cancer Res.*, **34**, 3245.

COPELAND, G.P., LEINSTER, S.J., DAVIS, J.C. & HEPKIN, L.J. (1987). Insulin resistance in patients with cholesterol cancer. *Br. J. Surg.*, **74**, 1031.

DE WAARD, F. (1975). Breast cancer incidence and nutritional status with particular reference to body weight and height. *Cancer Res.*, **35**, 3351.

DRASAR, B.S. & IRVING, D. (1973). Environmental factors and cancer of colon and breast. *Br. J. Cancer*, **27**, 167.

DYER, A.R., STAMLER, J., OGLESBY, P. & 7 others (1981). Serum cholesterol and risk of death from cancer and other causes in three Chicago-epidemiological studies. *J. Chronic Dis.*, **34**, 249.

FERNLEIB, M. (1983). Review of the epidimiological evidence for a possible relationship between hypocholesterolemia and cancer. *Cancer Res.*, **43**, 2503.

FUNG, K.P., CHAN, T.W. & CHOY, Y.M. (1985). Suppression of Erlich ascites tumour growth in mice by starvation and streptozotocin induced diabetes. *Cancer Lett.*, **28**, 273.

GAMMELTOFT, S. (1984). Insulin receptors, binding kinetics and structure function relationship of insulin. *Physiol. Rev.*, **64**, 1321.

GORANSON, E.S., BOTHAM, F. & WILLM, M. (1954). Inhibition of growth of transplantable alloxinated Wistar rats. *Cancer Res.*, **14**, 730.

GORANSON, E.S. & TILSER, G.J. (1955). Studies on the relationship of alloxan-diabetes and tumor growth. *Cancer Res.*, **15**, 626.

GRAHAM, S. (1986). Hypothesis regarding caloric intake in cancer development. *Cancer*, **58**, 1814.

GREENBERG, N.J., TZAGROURNIS, M. & GRAVES, T.M. (1968). Stimulation of insulin secretion in man by medium chain triglycerides. *Med. Clin. Exp.*, **17**, 796.

HARAN-GERA, N., BEN YAACOV, M. & PELED, A. (1977). Immunologic characteristics in relation to high and low leukomogenic activity of radiation leukemic virus variants. *J. Immunol.*, **118**, 600.

HARPER, H.A. (1965). Metabolism of fat. In *Review of Physiological Chemistry*, Wong, R.K.L. & VanBruggen, J.T. (eds) p. 223. Los Altos, CA: Blackwell Scientific Publications.

HEMS, G. (1978). The contribution of diet and childbearing to breast cancer rates. *Br. J. Cancer*, **37**, 974.

HEUSON, J.C. & LEGROS, N. (1970). Effect of insulin and alloxan on growth of the mammary carcinoma *in vivo*. *Eur J. Cancer*, **6**, 349.

HEUSON, J.C., LEGROS, N. & HEIMANN, R. (1972). Influence of insulin administration on growth of 7,12 dimenthyl-benz(a)-anthracene mammary carcinoma in intact, oophorectomized and hypophysectomized rats. *Cancer Res.*, **32**, 233.

HINDS, M.W., KOLONEL, L.N., LEE, J. & HANKIN, J.H. (1982). Dietary cholesterol and lung cancer risk among men in Hawaii. *Am. J. Clin. Nutr.*, **37**, 192.

HOBBS, C.B. & MILLER, A.L. (1966). Review of endocrine syndromes associated with tumors of non endocrine origin. *J. Clin. Pathol.*, **19**, 119.

HYRAYAMA, T. (1978). Epidemiology of breast cancer with special reference to the role of diet. *Prev. Med.*, **7**, 173.

JEHL, J.A., MAYER, J. & MCKEE, R.W. (1955). Influence of the hereditary obese hyperglycemic syndrome of alloxan diabetes on the survival of mice with Erlich ascites carcinoma. *Cancer Res.*, **15**, 341.

KARK, J.D., SMITH, A.H. & HAMES, C.G. (1980). The relationship of serum cholesterol to incidence of cancer in Evans Country, Georgia. *J. Chronic Dis.* **33**, 311.

KESLER, I.I. (1970). Cancer mortality among diabetes. *J. Natl Inst.*, **44**, 683.

KOLONEL, L.N., HANKIN, J.H., LEE, J., CHU, S.Y., NOMURA, A.M.Y. & HINDS, M.W. (1981). Nutrients intake in relation to cancer incidence in Hawaii. *Br. J. Cancer*, **44**, 332.

LINSCHEER, W.G., SLONE, D. & CHALMERS, T.C. (1967). Effects of octanoids on serum levels of free fatty acids, insulin and glucose in patients with cirrhosis and in healthy volunteers. *Lancet*, **i**, 593.

LUNDHOLM, K., HOLM, G. & SCHRESTEN, T. (1978). Insulin resistance in patients with cancer. *Cancer Res.*, **38**, 4665.

LUPULESCU, A. (1983). Glucagon control of carcinogenesis. *Endocrinology*, **113**, 527.

MCMICHAEL, A.J., JENSEN, O.M. & PARKIN, M.D. (1984). Dietary and endogenous cholesterol and human cancer. *Epidemiol. Rev.*, **6**, 192.

MILLER, A.B., KELLY, A., CHOI, N.W. & 7 others (1978). A study of diet and breast cancer. *Am. J. Epidemiol.*, **107**, 499.

MOUNTJOY, K.G., FINLAY, G.J. & HOLDAWAY, I.M. (1987). Abnormal insulin reception down regulation and dissociation of down regulation from insulin action in cultured human tumor cells. *Cancer Res.*, **47**, 6500.

MOUNTJOY, K.G., HOLDAWAY, I.M. & FINLAY, G.J. (1983). Insulin receptor regulation in cultured human tumor cells. *Cancer Res.*, **43**, 4537.

NRC (1978). *Nutrients Requirements of Laboratory Animals*, 3rd edn. National Academy of Science: Washington, DC.

PAVELIC, K., BOLANCA, M., VICEK, N., PAVELIC, J., MAROTTI, T. & VUK-PAVOLIC, S. (1982*a*). Carcinomas of the cervix and corpus uteri in human: stage-dependent blood levels of substance (s) immunologically cross reactive with insulin. *J. Natl Cancer Inst.*, **68**, 891.

PAVELIC, K., ODAVIC, M., HRSAK, I. & VUK-PAVLOVIC, S. (1982*b*). Correlation of substance (s) immunologically cross-reactive with insulin in Hodgkin's lymphoma patients. *Cancer Lett.*, **17**, 81.

PAVELIC, K. & PAVELIC, M. (1981). Insulin and glucagon secretion by renal adenocarcinoma. *Cancer*, **48**, 98.

PAVELIC, K. & SLYJEPCEVIC, M. (1978). Growth of a thymoma in diabetic mice treated with insulin. *Eur. J. Cancer*, **14**, 675.

PAVELIC, K. SLYJEPCEVIC, M., PAVELIC, J. & 4 others (1979). Growth and treatment of Erlich tumor in mice with alloxan induced diabetes. *Cancer Res.*, **39**, 1807.

PAVELIC, K. & VUK-PAVLOVIC, S. (1983). C peptides does not parallel increases of serum levels of substances immunologically cross-reactive with insulin in non-Hodgkin's lymphoma patients. *Blood*, **61**, 925.

PENNOCK, C.A., MURPHY, D., SELLERS, J. & LONGDON, K.J. (1973). A comparison of autoanalyser methods for the determination of glucose in blood. *Clin. Chim. Acta*, **48**, 193.

PUCKITT, C.L. & SHAGLETON, W.W. (1972). The effect of induced diabetes on experimental tumor growth in mice. *Cancer Res.*, **32**, 789.

RERUP, C.C. (1968). Spontaneous remission of alloxan-diabetes in mice. *Diabetologia*, **4**, 312.

ROSE, G. & SHIPLEY, M.S. (1980). Plasma lipids and mortality: a source of error. *Lancet*, **i**, 523.

SALZBERG, D.A. & GRIFFIN, A.C. (1952). Inhibition of Azo dye carcinogenesis in the alloxan diabetic rat. *Cancer Res.*, **12**, 294.

SHAFIC, S.M. & LIOTTA, L.A. (1980). Formation of metastasis by human breast carcinoma cells MCA.7 in nude mice. *Cancer*, **11**, 81.

SHAPOT, V.S. (1972). Some biological aspects of the relationship between the tumor and the host. *Adv. Cancer Res.*, **15**, 253.

SIEDEL, J., HAGELE, E.O., ZIEGENHORN, J. & WAHLEFED, A.W. (1983). Reagent for the enzimatic determination of serum total cholesterol with improved lipolytic efficiency. *Clin. Chem.*, **29**, 1075.

SMITH, L.L., SMART, V.B. & ANSARI, G.A.S. (1979). Mutagenic cholesterol preparations. *Mutation Res.*, **68**, 23.

STOLAR, M.W. (1988). Atherosclerosis in diabetis. The role of hyperinsulinemia. *Metab. Clin. Exp.*, **37**, Suppl.1, 1.

SZEPSENWOL, J. (1966). Carcinogenic effect of cholesterol in mice. *Proc. Soc. Exp. Med. Biol.*, **121**, 168.

YAARI, S., GOLDBOURT, V., EVEN ZOHAR, S. & NEUFELD, H.N. (1981). Associations of serum high density lipoprotein and total cholesterol with total cardiovascular study of 10000 man. *Lancet*, **i**, 1011.

YOSHINOBU, S., KASAI, K., HIRAIVA, M. & 4 others (1985). Monolayer cell culture of human insulinoma and in vitro studies of regulatory mechanisms on insulin release. *Dokkyo J. Med. Sci.*, **12**, 149.